# Investigation of a Novel Deep Learning-Based Computed Tomography Perfusion Mapping Framework for Functional Lung Avoidance Radiotherapy

**DOI:** 10.3389/fonc.2021.644703

**Published:** 2021-03-24

**Authors:** Ge Ren, Sai-kit Lam, Jiang Zhang, Haonan Xiao, Andy Lai-yin Cheung, Wai-yin Ho, Jing Qin, Jing Cai

**Affiliations:** ^1^Department of Health Technology and Informatics, The Hong Kong Polytechnic University, Hong Kong, Hong Kong; ^2^Department of Nuclear Medicine, Queen Mary Hospital, Hong Kong, Hong Kong; ^3^School of Nursing, The Hong Kong Polytechnic University, Hong Kong, Hong Kong

**Keywords:** perfusion imaging, lung function imaging, deep learning, perfusion synthesis, CT based image analysis, functional lung avoidance radiation therapy

## Abstract

Functional lung avoidance radiation therapy aims to minimize dose delivery to the normal lung tissue while favoring dose deposition in the defective lung tissue based on the regional function information. However, the clinical acquisition of pulmonary functional images is resource-demanding, inconvenient, and technically challenging. This study aims to investigate the deep learning-based lung functional image synthesis from the CT domain. Forty-two pulmonary macro-aggregated albumin SPECT/CT perfusion scans were retrospectively collected from the hospital. A deep learning-based framework (including image preparation, image processing, and proposed convolutional neural network) was adopted to extract features from 3D CT images and synthesize perfusion as estimations of regional lung function. Ablation experiments were performed to assess the effects of each framework component by removing each element of the framework and analyzing the testing performances. Major results showed that the removal of the CT contrast enhancement component in the image processing resulted in the largest drop in framework performance, compared to the optimal performance (~12%). In the CNN part, all the three components (residual module, ROI attention, and skip attention) were approximately equally important to the framework performance; removing one of them resulted in a 3–5% decline in performance. The proposed CNN improved ~4% overall performance and ~350% computational efficiency, compared to the U-Net model. The deep convolutional neural network, in conjunction with image processing for feature enhancement, is capable of feature extraction from CT images for pulmonary perfusion synthesis. In the proposed framework, image processing, especially CT contrast enhancement, plays a crucial role in the perfusion synthesis. This CTPM framework provides insights for relevant research studies in the future and enables other researchers to leverage for the development of optimized CNN models for functional lung avoidance radiation therapy.

## Introduction

Perfusion sustains the normal pulmonary gas exchange and is one of the main functions of the lungs (1). Lung perfusion imaging measures the blood circulation within the lung and is commonly used in the clinic to present the regional functional information (2). In radiation therapy of lung cancer, lung perfusion images can be utilized as a predictor of regional lung function to guide functional lung avoidance radiotherapy (FLART) (3, 4). FLART is a developing technique that avoids irradiating highly functional lung regions based on the regional function information, holding great promises to reduce radiation-induced lung injury and improve the toxicity outcomes of lung cancer radiation therapy (RT) (5–7).

Several imaging modalities have been developed to generate pulmonary perfusion images. Nuclear medicine based SPECT perfusion is most commonly employed in clinical practice (8). Radioactive albumin, such as Technetium-99m-labeled macro-aggregated albumin (^99m^ Tc MAA), is injected into the patients prior to image acquisition. The signal delivered by the radioactive particles reflects the blood flow within the lung and is measured by a single-photon emission computed tomography (SPECT) scanner. However, SPECT perfusion requires the injection of radioactive particles and provides limited temporal resolution, rendering it less popular in most medical institutions. With the advancement of multi-voltages X-ray tubes, the dual-energy CT (DECT) also showed the ability to detect embolic pulmonary arterial vessel occlusion based on the material spectral property (9, 10). Although DECT offers an improved spatial resolution compared with the SPECT perfusion (11), it is of low accessibility in most hospitals worldwide. More importantly, it increases the amount the radiation exposure to patients.

In view of this, we proposed a novel deep learning-based framework for CT perfusion mapping (CTPM) to overcome these limitations based on the previous study (12). There are several rationales behind this CTPM-dedicated framework. First of all, CT scans contain high-resolution information and are widely accessible in most clinics, as it is routinely utilized for radiological assessment of diseases and RT treatment planning worldwide. Secondly, a variety of pulmonary diseases are generally manifested as intensity alterations in the lung parenchyma. For instance, the interstitial lung diseases related to inflammation and fibrosis of lung tissue can be interpreted from CT patterns, including reticulation, honeycombing, ground-glass opacity, consolidation, and micronodules (13). Thirdly, the multi-layered convolutional models have shown promising capability in various medical image synthesis tasks, such as medical image quality improvement (14, 15), MR-to-CT translation (16, 17), and super-resolution MRI images (18). These rationales have led to the hypothesis that deep learning-based features extracted from anatomic CT images contain a myriad of concealed physiologic and biologic information in relation to lung function, and hence it is feasible to convert anatomic CT scans into functional images for pulmonary perfusion. The benefit of deep learning-based CTPM can be further magnified when it comes to implementing FLART, because (i) it protects patients from receiving additional radiation dose, since no extra image tests would be required and (ii) it prevents patients from delaying commencement of RT treatment due to either the extra time or financial difficulties to take additional tests, since the deep-learning based methods are considered to be more time-efficient.

To the best of our knowledge, we were the first to demonstrate the feasibility of CT to perfusion translation. In our previous work, we concluded that our deep learning-based CTPM method yielded a moderate-to-high approximation to SPECT perfusion images. However, the principles of this CTPM method was not explored yet. The current study aims to comprehensively investigate and compare the impacts of different components of the deep learning-based framework through a series of ablation experiments, in which individual tested components were removed to study its impact on the framework performance. Our overarching purpose is to provide insights for relevant research studies in the future and enable scientists to be able to leverage the results of this study for the development of optimized deep learning models for CTPM.

## Materials and Methods

### Dataset and Study Design

In this study, 42 pulmonary perfusion MAA SPECT/CT scans were retrospectively collected from Queen Mary Hospital. The detailed patient characteristics are listed in **Table 1**. The use of the image data was approved by the Institutional Review Boards (IRB) of the University of Hong Kong/Hospital Authority Hong Kong West Cluster. Each CT slice was collected as a 512 × 512 matrix with a pixel spacing of 0.977 × 0.977 mm^2^, and the axial spacing is 1.25 mm. Each SPECT volume was constructed as a 128 × 128 × 128 matrix with 4.42 × 4.42 × 4.42 mm^3^ voxel size. For consistency, all the image pairs were resampled to a voxel size of 1 × 1 × 1 mm^3^ for the following study.

**Table 1:** Patient characteristics.

		Number	Percent
Sex	Male	15	35.7%
	Female	27	64.3%
Age	Mean ± SD	67 ± 14	
Diagnosis	Pulmonary hypertension	14	33.3%
	Lung carcinoma	10	23.8%
	Pulmonary embolism	5	11.9%
	Systemic lupus erythematosus	2	4.7%
	Chest pain	2	4.7%
	Others	9	21.4%

*Others: Pulmonary fibrosis, hypoxia, congestive heart failure, aortic dissection, pulmonary arteriovenous malformation, hepatopulmonary syndrome, shortness of breath, hypoplastic right ling, normal.

**Figure 1:**
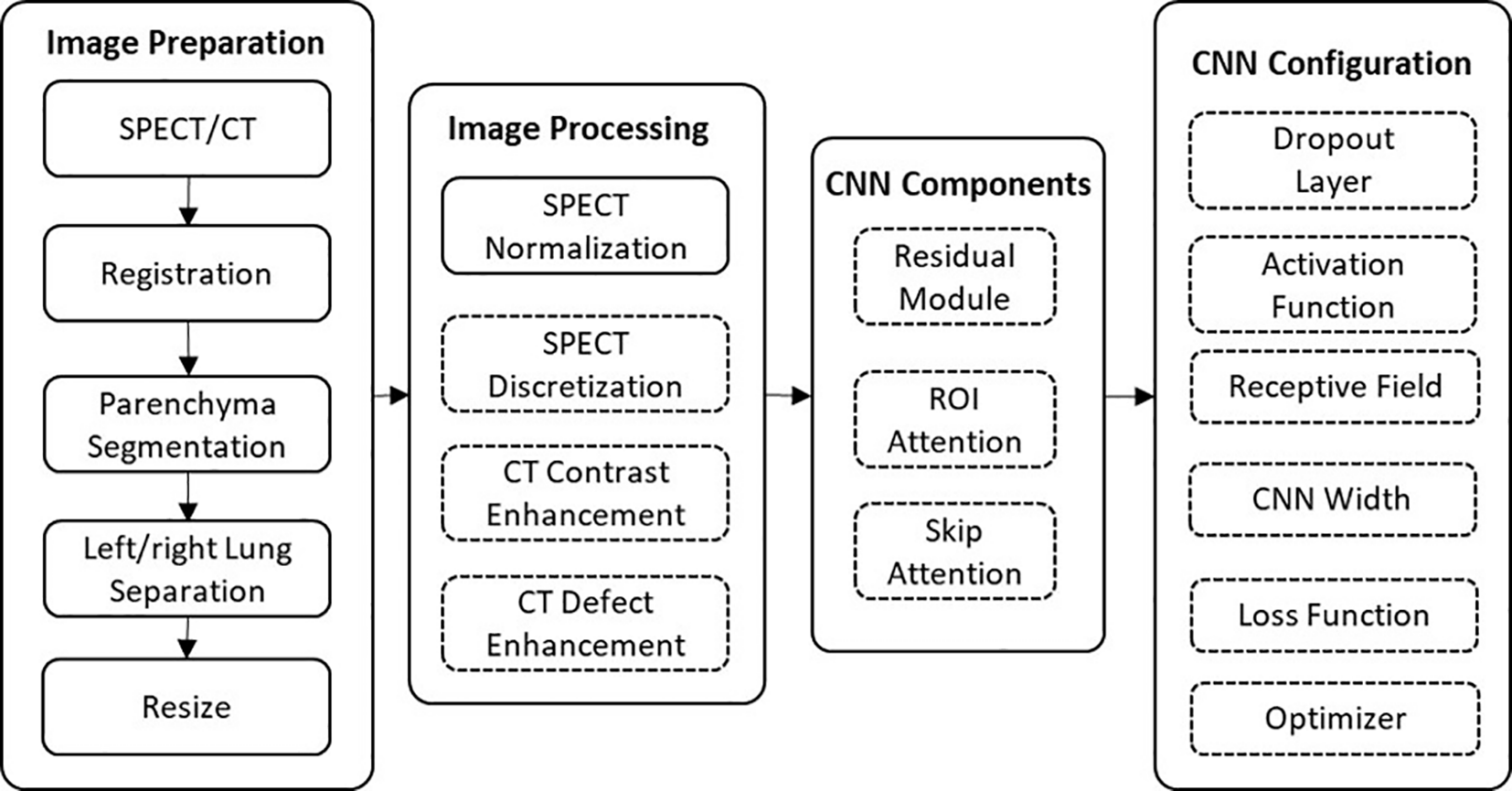
The workflow of this framework. The effects of the components in the dashed outlined box were evaluated using ablation experiments.

These patients were randomly split into a training group (31 patients) and a testing group (11 patients). The CT and SPECT images of each patient went through image preparation and image processing prior to model training and testing. In image preparation, the left and right lungs were separated to augment the dataset size to 84 (62 for training and 22 for testing). The effectiveness of different components and parameters of this framework were analyzed using a series of ablation experiments (**Figure 1**). The CTPM generated perfusion images were compared with the processed SPECT perfusion label images using the structural similarity (SSIM) and correlation coefficient (R). After deciding the final framework, the generated CTPM perfusion images were recovered to the original size with post-processing and compared with the SPECT perfusion in the original size using the Dice similarity coefficient (DSC) in clinical relevance.

### Image Preparation

Image preparation consists of a series of morphological operations to decrease computational consumption and remove noise. Firstly, a parenchyma mask was automatically generated in CT images using the Chest Imaging Platform, which is an open-source library for quantitative chest imaging (19). The trachea and tumor regions were excluded from the mask. This mask was subsequently applied to the SPECT and CT images to segment the parenchyma volume. In order to reduce the memory cost of 3D image calculation, the left and right lungs were separately analyzed in our study, enabling mitigation of the image complexity and doubling the sample size. To further minimize the consumption of the computational power, the separated parenchyma volumes were cropped to include only the lung and resized to 128 × 64 × 64 voxels.

### Image Processing

Image processing aims to enhance the robustness of CT extracted features and standardize SPECT perfusion images to be suitable labels for CNN mapping. The down-sampled CT and SPECT images were standardized using CT enhancement, SPECT normalization/discretization, respectively.

#### SPECT Normalization and Discretization

The signal intensity of pulmonary SPECT perfusion is strongly affected by both the breathing pattern and blood flow condition of an individual. As a result, the SPECT value may vary significantly from patient to patient, which decreases the supervision function of the SPECT. To address this problem, SPECT image normalization was commonly used to reduce the influence of individual conditions (20–22). In this study, the SPECT image was first normalized using the values in normal functional regions, and then discretized into 11 uniform values ranging from 0 to 1 to reduce the fluctuation noise in SPECT. Data discretization is another commonly used technique for machine learnings (23). This technique is used to decrease the noise-induced variance of the extracted features and speeding up the modeling convergence. Image discretization has been used in PET ventilation images of lung functional imaging to mitigate small noise fluctuations (24). In this study, the processed SPECT images were used as the label for model training. The normalization and discretization procedures are described by Eq. 1:


(1) $$SPECT(x,y,z)'=discritize\left(\frac{SPECT(x,y,z)}{HIR}\right)$$

where *SPECT*(*x,y,z*) is the SPECT value at the location of (*x,y,z*). *SPECT*(*x,y,z*)*'* is the processed SPECT value. *HIR* is the high-intensity region value, which was set as the 75^th^ percentile of the pixel values in this study since this is close to the normal lung perfusion (25). *discretize* indicates the above described SPECT discretization.

#### CT Enhancement

This enhancement aims to improve the intensity difference between low/high functional regions on CT images, including CT contrast enhancement and CT defect enhancement. CT contrast enhancement is achieved based on histogram equalization, which can effectively display different regions of pulmonary CT images (26). The down-sampled CT images were equalized within Hounsfield Unit (HU) values from −1,000 to −300. Contrast enhancement is followed by applying filters, which has been proven to improve the correlation between CT and lung functional images (21). A median filter with a kernel size of 10 pixels was subsequently applied to enhance the signal in low functional regions and to reduce the noise fluctuation. This was followed by using a uniform filter with a kernel size of five pixels for further noise reduction. These CT enhancement procedures were formulated by Eq. 2:


(2) $$CT(x,y,z)'=filtering(\frac{chf[CT(x,y,z)]-chf_{-1000}}{chf_{-300}-chf_{-1000}})$$

where *CT*(*x,y,z*) is the original HU value of (*x,y,z*). *CT*(*x,y,z*)*'* is the enhanced CT image. The cumulative histogram function (*chf*) calculates the cumulative counts for each HU bin. *chf*_−1000_ is the *chf* in value of −1000 and *chf*_−300_ is for −300. The value of outliers was replaced with the threshold value. *filtering* indicates the above-described filters.

### CNN Architecture

A 3D CNN model usually generates an exceeding number of parameters, which cause high memory consumption and increased likelihood of model overfitting. Since the mapping correlation between CT and SPECT perfusion have not been explored before this study, we proposed and constructed an adjustable CNN model for CT-to-perfusion translations. The proposed CNN follows a 3D encoding-decoding structure. Two skip attention modules similar to attention U-Net (27) were used to translate the local details captured in the feature maps from the contraction path into the expansion path. The convolution layers capture the hierarchical texture features of the input. To increase the size of the receptive field, three 2 × 2 × 2 stride convolutions and convolution layers with a filter size of 5 × 5 × 5 were used. Each convolution is followed by batch normalization, parametric rectified linear unit (PReLU), and a dropout layer. At the last layer, a Sigmoid function sums up the results of the previous layers and maps them to the range of [0,1] as the final prediction values. In order to determine the optimal architecture, the significance of the following components and configurations on framework performance was tested with a series of experiments.

#### CNN Components

**Figure 2:**
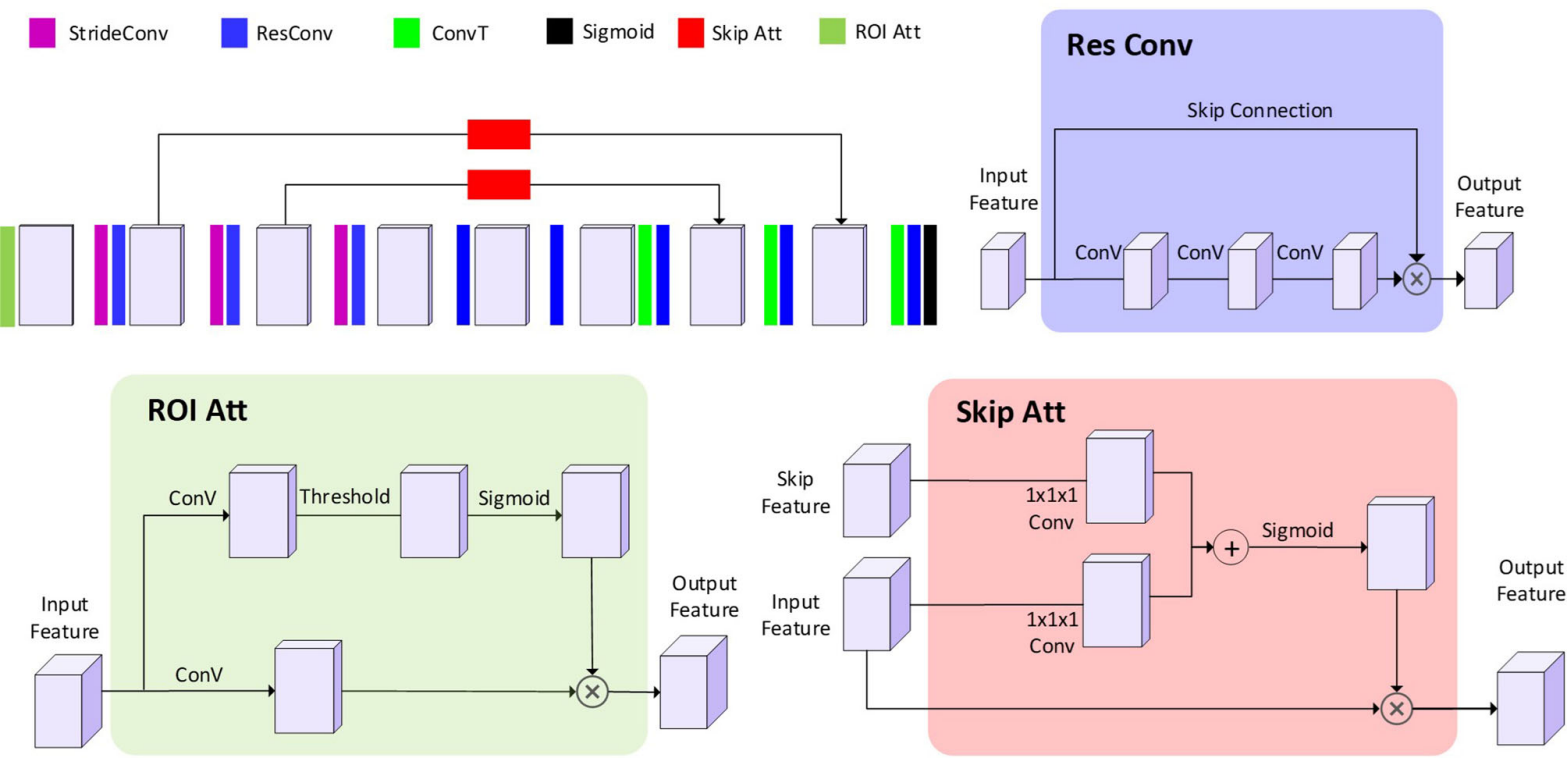
The architecture of the proposed CNN model. The 3D blocks indicate the feature map. StrideConv was short for stride convolution. ResConv is short for residual convolution. ConvT is short for convolution transpose. Sigmoid is short for the sigmoid layer. Skip Att is short for the skip attention module. ROI Att is short for ROI attention.

The impacts of three components (residual module, ROI attention, and skip attention) were tested using ablation experiments. Residual modules have shown to be capable of speeding up the convergence (28). In our model, eight residual modules were designed in the middle of the network (**Figure 2**). The ROI attention module was configurated on the first layer to steer the data learning process focusing on the parenchyma volumes, avoiding degraded coverage of loss function owing to the meaningless learning on the image background (*i.e.*, non-parenchyma region). The skip attention module has been found to benefit target structure attention (29). Because low functional regions normally are smaller than functional regions, two skip attention connections were used to help focus on the low functional region.

#### CNN Configuration

Multiple elements were particularly designed to minimize the risk of model overfitting. Our proposed model uses dropout layers and early stop to alleviate the likelihood of overfitting. Each convolution and activation function are followed by a dropout layer with a dropout rate in the range of [0, 0.1, 0.2, 0.3, 0.4, 0.5].

Although a large filter size can facilitate global texture feature extraction in the receptive field, it simultaneously increases the number of parameters and decreases the learning ability of the model. In order to explore the effect of filter size on the learning ability, 5 × 5 × 5 convolution filters and 3 × 3 × 3 convolution filters were tested. The number of CNN parameters was controlled by the CNN width. In this study, a series of CNN widths (3 × 32, 4 × 32 and 5 × 32) were also tested to optimize the fitting power of the CNN model.

ReLU activation functions have been proven to speed up the training process compared to classic sigmoid alternatives. In this study, a parametric ReLU (PReLU) (30) was also developed from ReLU, as formulated in Eq. 3:


(3) $$PReLU(x)=\{\begin{array}{c} \;x,x>0\\ ax,x\leq 0 \end{array}$$

where *a* is a learnable parameter. This loss function does not zero out the negative input so that a small gradient is allowed when the unit is not active. For the encoding and decoding structure, the PReLU was used after each convolution layer. LeakyReLU is PReLU with a fixed slop. The performances of PReLU, LeakyReLU (slope = 0.5), and ReLU were tested in the validation group.

### Evaluation Metrics

In the tuning of the model, the framework performance of each experiment was evaluated using an analysis metric between the CT perfusion mapping (CTPM) perfusion and processed SPECT perfusion. The analysis metric was the sum-up of R and SSIM, accounting for stassssstistical and perceptual image similarity, respectively. The Spearman correlation coefficient was defined using the following equation:


(4) $$R=\frac{\Sigma_{i=1}^{N}[(y_{i}-\bar{y})\cdot (p_{i}-\bar{p})]}{\sqrt{\Sigma_{i=1}^{N}{\left(y_{i}-\bar{y}\right)}^{2}}\sqrt{\Sigma_{i=1}^{N}{\left(p_{i}-\bar{p}\right)}^{2}}}$$

where *p_i_* and *y_i_* indicate the predicted and ground-truth perfusion value at voxel *i*. *N* denotes the total number of non-zero voxels. *R* is within the range of [−1,1] and represents the intensity monotonicity of spatially correlated voxels.

The SSIM is based on the comparison of luminance term, contrast term, and structural term between the samples of generated perfusion and SPECT perfusion (31). The overall SSIM is a multiplicative combination of the three terms:


(5) $$SSIM=\frac{2\mu_{y}\mu_{p}+C_{1}}{{\mu_{y}}^{2}+{\mu_{p}}^{2}+C_{1}}\cdot \frac{2\sigma_{yp}+C_{2}}{\sigma_{y}^{2}+\sigma_{p}^{2}+C_{2}}$$

where *µ_y_*, *µ_p_*, *σ_y_*, *σ_p_*, and *σ_yp_* are the local means, standard deviations, and cross-variance for ground-truth image *y* and predicted image *p C*_1_ = (*k*_1_*L*)^2^, C_2_ = (*k*_2_*L*)^2^ are the two variables that stabilize the divisions when the denominators are too small. *L* is the dynamic range of the pixel values. By default, *k*_1_ is set to 0.01, and *k*_2_ is set to 0.03.

### Overall Performance Analysis

After deciding the final framework, the potential applicability in FLART was evaluated. In FLART, the lung will be divided into different functional regions based on the functional image information. In this study, the generated CTPM perfusion images were firstly resampled to the original SPECT perfusion size and divided into low and high functional lung regions. These two regions in CTPM were compared with that in original SPECT perfusion using the Dice similarity coefficient (Equation 6). This approach was used in the earlier ventilation/perfusion-SPECT study (20) and Galligas-positron emission tomography study (32). The low/high functional threshold was set to 0.66, which has been used in a lung ventilation study (33) and suggested for FLART plan optimization (34).


(6) $$DSC=\frac{2*|p\cap y|}{\left|p|+|y\right|}$$

where *p* is low/high functional regions in the resampled CTPM perfusion and *y* is the normalized SPECT perfusion.

### Implementation

All the framework and analysis were coded in python. The CNNs worked based on the Pytorch 1.1 framework. The initialization of the convolutional layers of the CNNs was configurated using the Kaiming Uniform method (35). each layer was updated using error backpropagation with an adaptive moment estimation optimizer (ADAM), which is a first-order gradient-based algorithm designed for the optimization of stochastic objective functions with adaptive weight up with adaptive weight updates based on lower-order moments. The binary cross-entropy (BCE) was used as the loss function to evaluate the performance. The number of training epochs was set as 500, where the loss function changed less than 0.2% for continued five epochs in the pilot study. All the experiments were performed using a workstation with CPU Intel Core i7-8700 @ 3.2GHz, GPU NVIDIA GTX 2080 TI with 11GB memory, and 32 GB of RAM.

## Results

### Effects of Image Processing

The effects of image processing were firstly analyzed by removing different processing procedures using the proposed CNN model in **Table 2**. Removing the CT contrast enhancement step, the overall performance of the framework was jeopardized by ~11%. Removing the median filter degraded the overall performance by ~5%, and removing the uniform filter affected the overall performance by ~2% reduction. Removing SPECT discretization decreased the correlation by 3% and increased the SSIM by ~4%, resulting in an approximately equal overall performance.

**Table 2:** The effects of steps in the image processing pipeline.

Ablation experiments	*R*		*SSIM*		*SSIM*+*R*	Percent difference
	Average	SD	Average	SD		
CTPM	0.6655	0.1351	0.7077	0.0740	1.3732	/
CTPM-disc.	0.6468	0.1697	0.7327	0.0899	1.3795	0.46%
CTPM-contr.	0.5702	0.2157	0.6520	0.0850	1.2222	−11.00%
CTPM-med.	0.6158	0.1869	0.6869	0.0798	1.3027	−5.13%
CTPM-uni.	0.6473	0.1535	0.6939	0.0662	1.3412	−2.33%

**Figure 3:**
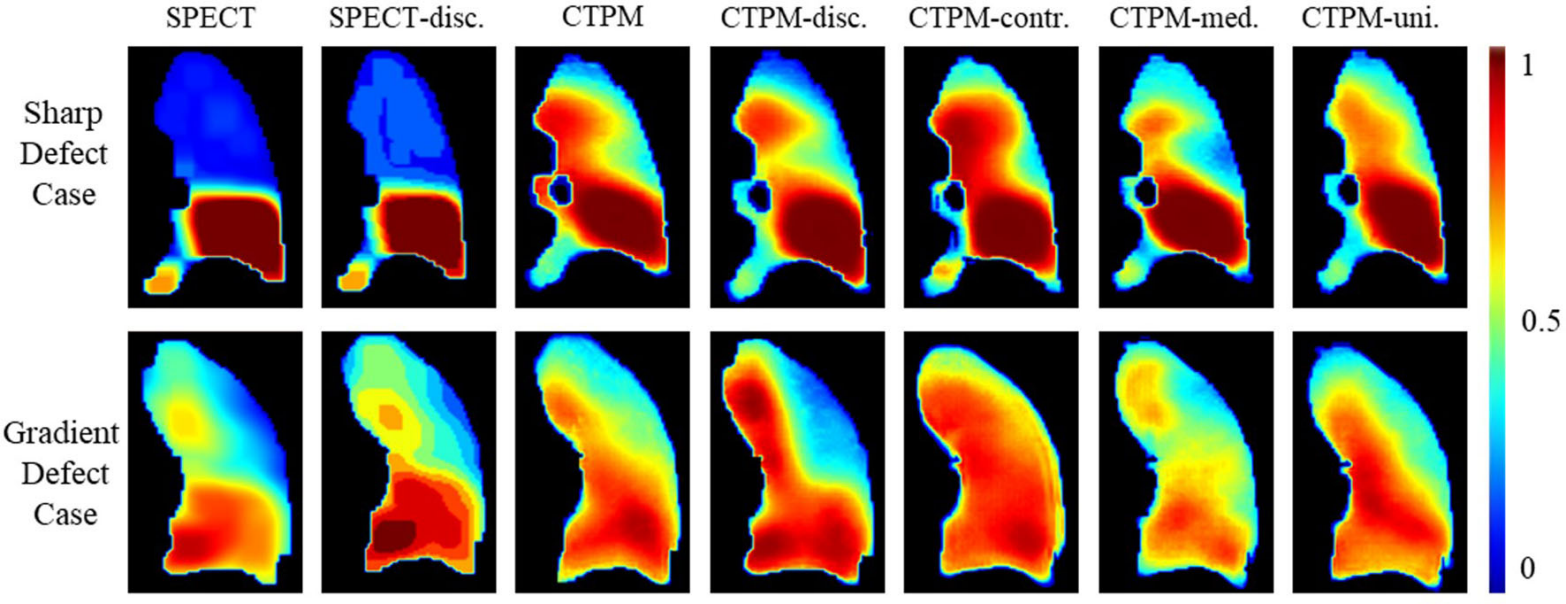
Illustration of the effects of different image processing steps in coronal view. Two representative cases with the sharp low functional and gradient low functional were visualized for comparison. The SPECT perfusion was normalized using the approach described in the method section. The color means the perfusion level. SPECT-disc. was the discretized label. CTPM was generated using the proposed setting. The following CTPMs were generated with five ablation scenarios: disc.—remove label discretization; contr.—remove CT contrast enhancement; med. —remove the median filter in CT low functional enhancement; uni.—remove the uniform filter in CT low functional enhancement.

To further explore the effects on images, two representing cases with a sharp changing defect and a gradient changing defect were visualized for qualitative analysis (**Figure 3**). For the sharp changing case, predicted perfusions of all the scenarios have a relatively high value (~0.7) on the upper lobe. The scenario of CTPM_-med._ generates the largest low functional region in this group. For the gradient changing case, the scenario CTPM generates the largest low functional region. For both cases, removing the CT contrast enhancement causes an overestimation of the value in the low functional region.

### Effects of CNN Structures

**Figure 4:**
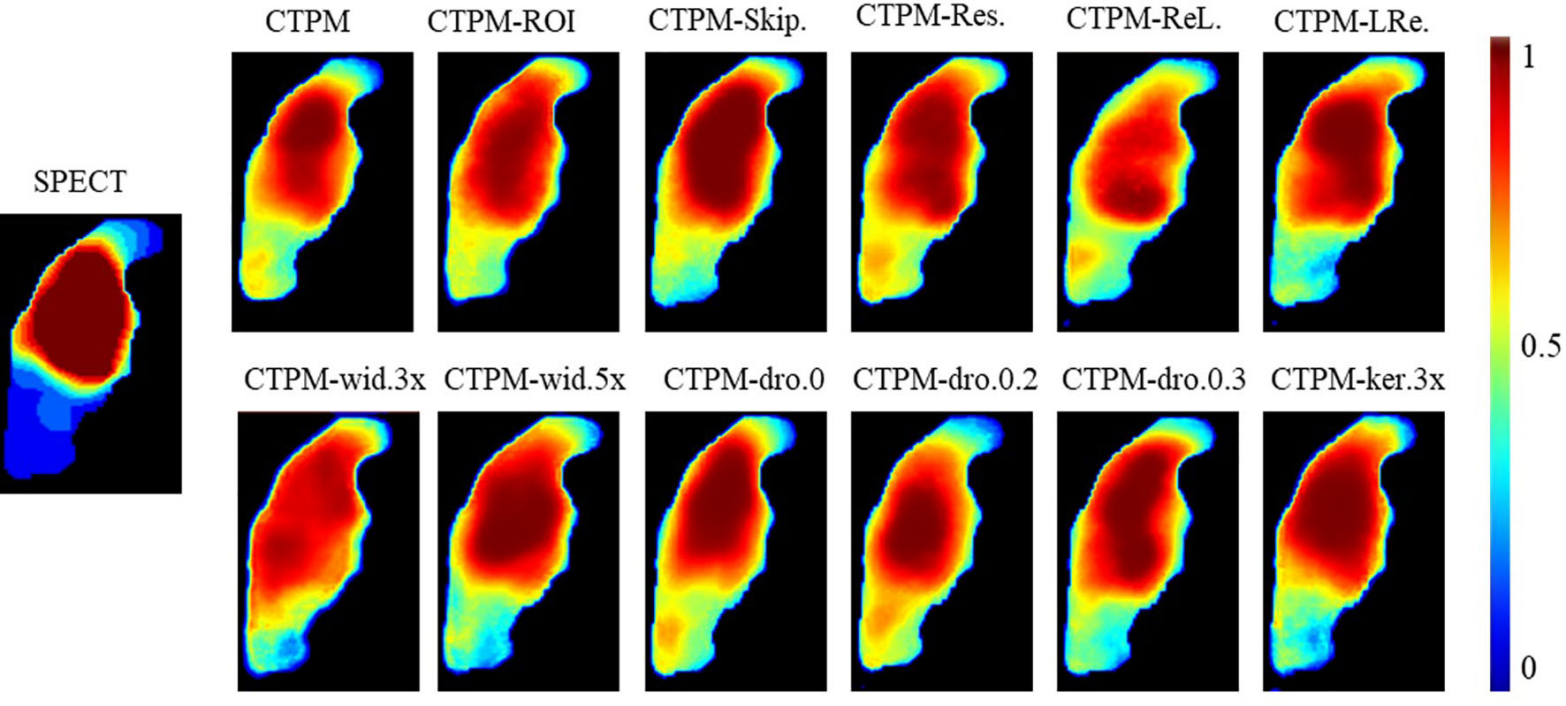
Illustration of the effect of varying CNN components and configurations of the representing case in coronal view. CTPM was generated using the proposed setting. The following CTPMs were generated in different CNN components and configurations: ROI.—remove ROI attention module, Skip.—remove skip attention module; Res.—remove residual module; ReL. – use ReLU instead of PReLU; LRe. – use LReLU instead of PReLU; wid.—with CNN width of 3, 4, or 5 times; dro—with a dropout rate of 0, 0.2, or 0.3; ker 3×—use kernel size of 3 × 3 × 3 install of 5 × 5 × 5.

The proposed components and configurations, presented in bold in **Table 3**, yielded a performance score of 1.3703. By altering the maximum layer width of CNN, we can infer that optimal architecture has 128 filters (max CNN width 4 × 32) in the middle of the network. Increasing the kernel size from 3 × 3 × 3 to 5 × 5 × 5 resulted in a 3% improvement in performance. Using the dropout layer with a parameter of 0.1 increased the performance by 4.3% compared with the model without the dropout layer. Compared with ReLU or LeakyReLU (0.5), the performance with PReLU increased by 5.9 and 2.4%, respectively. The ROI attention, skip attention, and the residual module increased the performance by 4.4, 3.1, and 5.4%, respectively. In the visualization analysis, predicted perfusions of all CTPM scenarios are able to predict the low functional region (**Figure 4**).

**Table 3:** Performance of the proposed network with different CNN components and configurations.

CNN Width	Kernel Size	Dropout Rate	ROI Attention	Skip Attention	Residual Module	Activation Function	*SSIM*	*R*	*SSIM*+*R*	Percent Difference
3 × 32	5 × 5 ×5	0.1	1	1	1	PReLU	0.6757	0.6305	1.3062	-4.68
**4 × 32**	**5 × 5 × 5**	**0.1**	**1**	**1**	**1**	**PReLU**	**0.7052**	**0.6651**	**1.3703**	**0**
5 × 32	5 × 5 × 5	0.1	1	1	1	PReLU	0.6831	0.6272	1.3103	-4.38
4 × 32	5 × 5 × 5	0	1	1	1	PReLU	0.6884	0.6236	1.3120	-4.25
4 × 32	5 × 5 × 5	0.2	1	1	1	PReLU	0.7014	0.6634	1.3648	-0.40
4 × 32	5 × 5 × 5	0.3	1	1	1	PReLU	0.7006	0.6519	1.3525	-1.30
4 × 32	3 × 3 × 3	0.1	1	1	1	PReLU	0.6867	0.6361	1.3228	-3.47
4 × 32	5 × 5 × 5	0.1	1	1	1	LReLU	0.6911	0.6458	1.3369	-2.44
4 × 32	5 × 5 × 5	0.1	1	1	1	ReLU	0.6882	0.6012	1.2894	-5.90
4×32	5×5×5	0.1	/	1	1	PReLU	0.6819	0.6276	1.3095	-4.44
4×32	5×5×5	0.1	1	/	1	PReLU	0.6879	0.6400	1.3279	-3.09
4×32	5×5×5	0.1	1	1	/	PReLU	0.6877	0.6085	1.2962	-5.41

The bold values indicates the optimal configurations.

### Overall Performance Analysis

**Figure 5:**
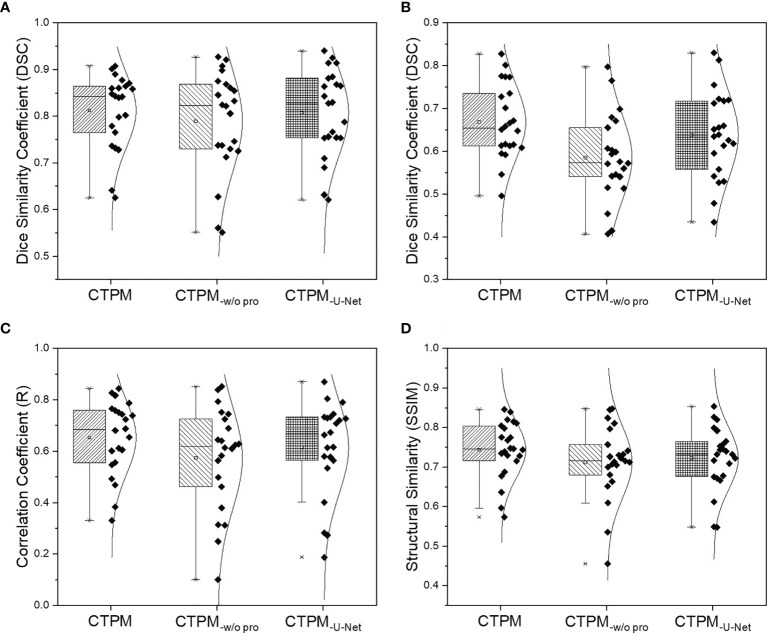
Overall performance analysis of the CTPM framework for the testing group. **(A)** DSC of the high functional lung. **(B)** DSC of the low functional lung. **(C)** Correlation coefficient. **(D)** Structural similarity. CTPM_-w/o pro_ indicates prediction without image processing. CTPM_-U-Net_ indicates prediction using U-Net.

**Figure 6:**
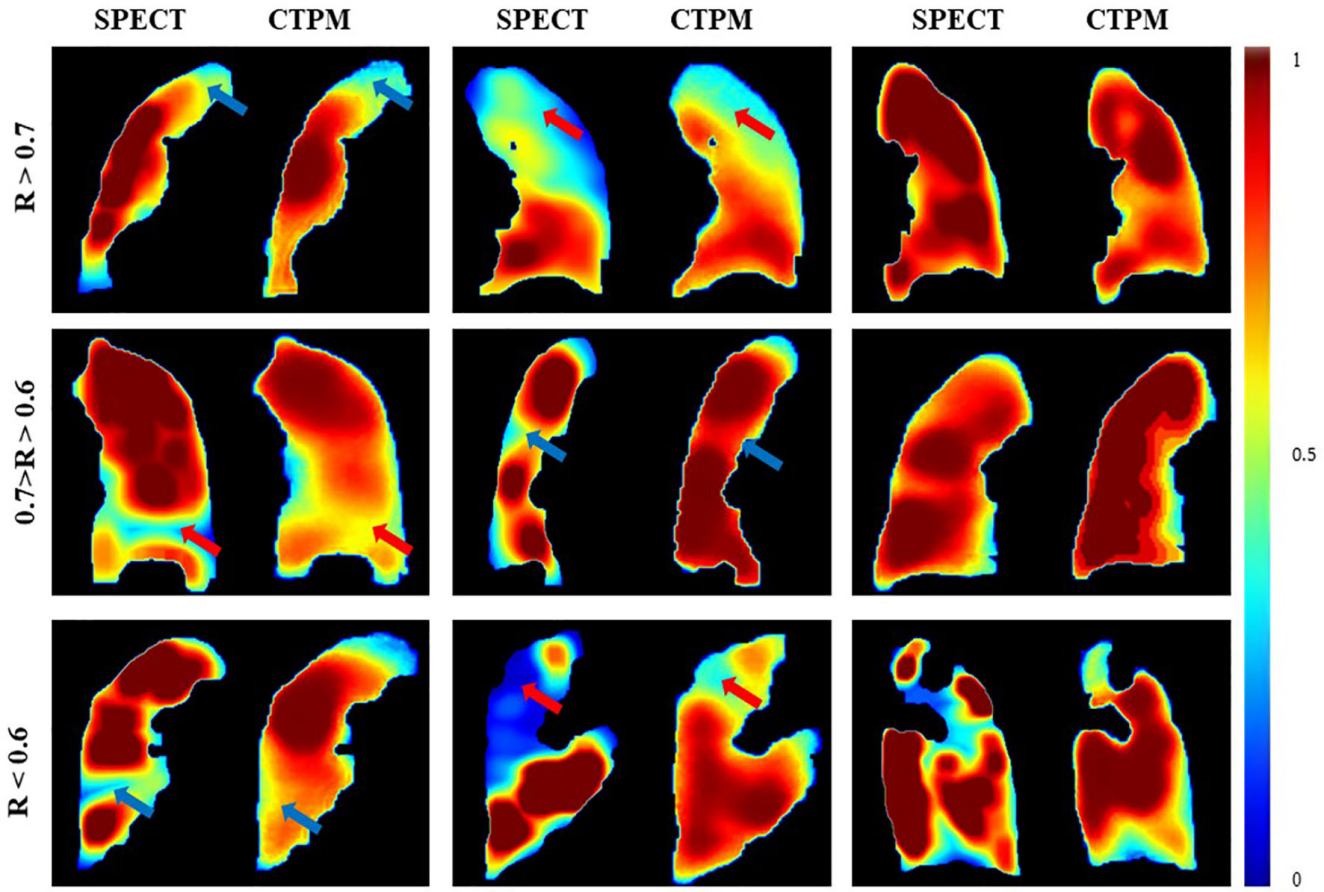
Comparison of the SPECT perfusion and CTPM perfusion of representative cases in the testing group. The arrows point to the primary low functional regions.

The performance of the final CTPM framework was illustrated in **Figure 5**. For the testing group, the proposed CTPM framework achieved an average DSC value of 0.8120 ± 0.0789 for high functional lung, average DSC value of 0.6682 ± 0.0867 for low functional lung, the average R value of 0.6534 ± 0.1432, and average SSIM value of 0.7437 ± 0.0739. The normalized SPECT perfusion and CTPM perfusion of representative cases in the testing group were visualized in coronal views in **Figure 6** for qualitative evaluations. In the 22 testing cases, 10 cases (45.5%) have correlation values larger than 0.7; six cases (27.3%) have correlation values between 0.6 and 0.7; six cases (27.3%) have correlation values less than 0.6.

Compared with the widely used U-Net using identical image processing procedures. The proposed perfusion mapping CNN model outperformed the U-Net by 0.5% (average DSC value of high functional lung), 4.8% (average DSC value of low functional lung), 6.3% (average R value), and 3.0% (average SSIM value). The proposed CNN model can significantly reduce the computational time (2.5 h) compared with U-Net (11.3 h). Additionally, removing the image processing procedures in the framework decreased the four metrics by 2.8, 12.4, 12.2, and 4.2%, respectively.

## Discussion

In this study, we analyzed the impact of the main components and configurations in a deep learning-based framework that estimates the 3D lung perfusion from the CT domain. The proposed deep convolutional neural network, in conjunction with a series of image preparation and image processing, is capable of feature extraction from the CT domain for perfusion synthesis. In the proposed framework, image processing, especially CT contrast enhancement, plays a crucial role in the perfusion synthesis. Before our study, most of the existing CT based pulmonary function studies used (24, 36, 37) deformable image registration (DIR) algorithms to derive ventilation in view of that both pulmonary ventilation and perfusion are correlated with the radiotherapy-induced pneumonitis (38). However, the accuracy of DIR based lung function mapping methods may be variable in different DIR algorithms and settings (39). Another HU number-based method estimates air and tissue densities from average CT, and then calculates the physiological ventilation in terms of the regional product of the densities, reaching a correlation score of 0.50 ± 0.17 with the ground-truth (32). Our optimized CNN model can achieve a voxel-wise agreement (R value of 0.6534 ± 0.1432, and SSIM value of 0.7437 ± 0.0739) and a regional similarity (DSC value of 0.8120 ± 0.0789 for high functional lung, DSC value of 0.6682 ± 0.0867 for low functional lung) with the corresponding reference SPECT perfusion. The regional information holds great promise for functional lung avoidance radiation therapy.

**Figure 7:**
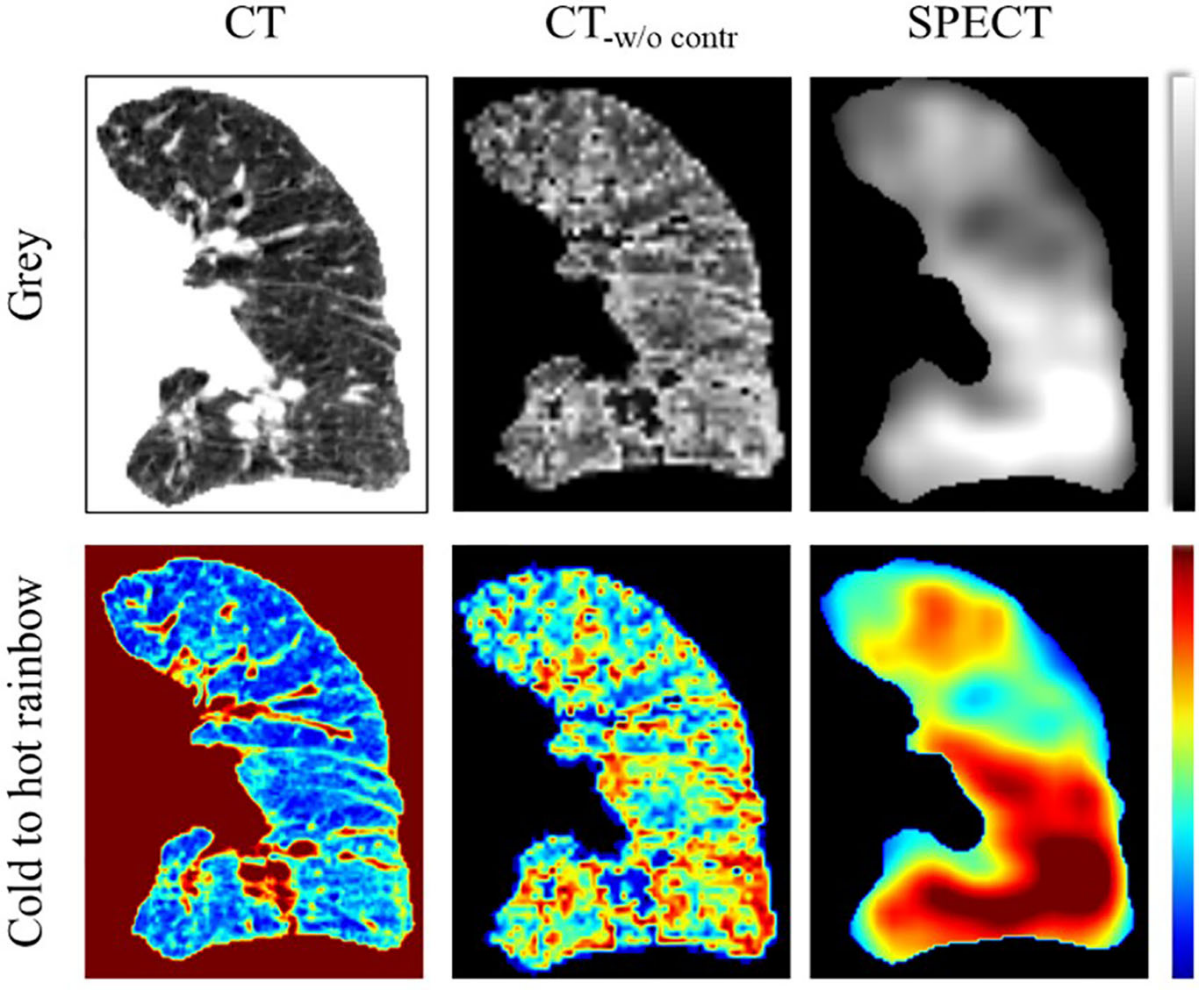
Illustration of the CT contrast enhancement of a representative case in coronal view. The CT image was visualized in the range of −1000 to 0. The SPECT image was visualized in the range of 0 to 800. CT_-w/o contr_ is the CT image after contrast enhancement.

In the CTPM framework, the removal of the CT contrast enhancement in the image processing part has the largest impact on perfusion prediction. In quantitative analysis, the framework performance dropped by ~11% as compared to the optimal performance. In the visualization analysis, the scenario without CT contrast enhancement results in all predictions as a high functional region. One possible explanation is that the low functional lung locates in a small range of HU values, in which the features are hard to be extracted by the CNN models. After contrast enhancement, the difference between high/low functional regions was improved (**Figure 7**) and became more “prominent” to the CNN models. As a result, CT contrast enhancement plays a critically important role in this translation. In the contrast enhancement, the setting of some parameters, such as the filter size, still relies on the model tunning on the validation set. In the future, it would be better to integrate these filters into the neural network to eliminate the bias caused by humans. This is feasible in principle because CNN is also composed of multi-layers of filters for feature extraction and signal transformation (40). However, this integration should be under the premise of a large cohort of patients since a CNN with more functions indicates more parameters to train.

**Figure 8:**
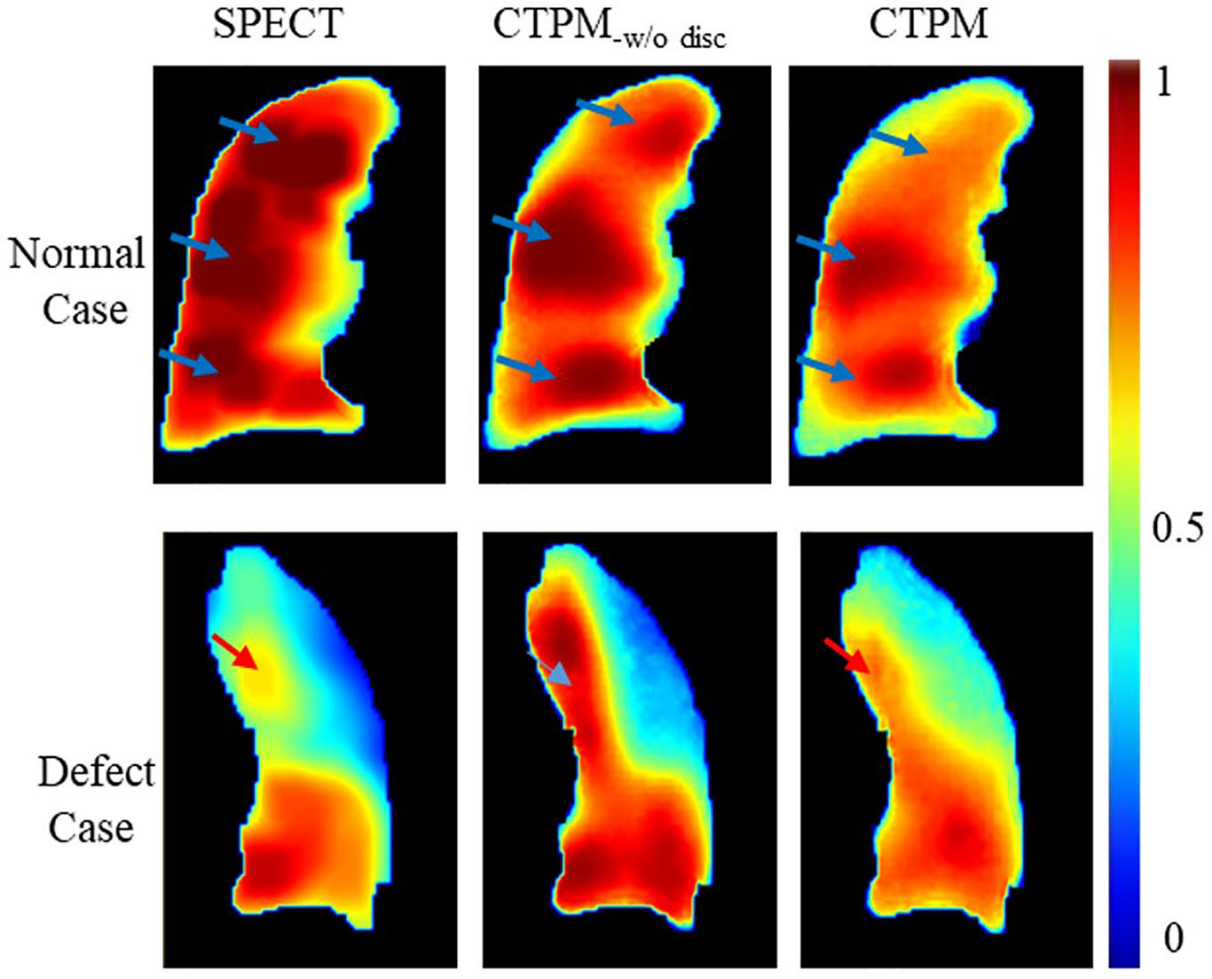
Effects of the SPECT discretization. CTPM_-w/o disc_ indicates prediction without SPECT discretization procedure.

Interestingly, SPECT discretization can improve R but decrease SSIM. Here we still recommend the SPECT discretization. In qualitative analysis, higher SSIM links to improved accuracy of the normal cases while higher R corresponds to enhanced accuracy of low functional cases (**Figure 8**). For the normal case, three high-value regions can be observed on one coronal slice of both continuous and discretized SPECT. The model trained with continuous labels can successfully predict the three peak regions, while only two of them can be observed on the prediction image from the discretized model. However, for the low functional case, the high function region in the upper lobe (red arrow) has much higher signals in CTPM_-disc_. In contrast, the perfusion using discretized label shares more similarity with the SPECT perfusion.

The CNN components and configurations also have important effects on the framework performance. All the three components (residual module, ROI attention, and skip attention) were approximately equally important to the framework performance; removing either one of them resulted in a 3–5% decline in performance. The ROI attention module can improve the performance by 5.7%, indicating the importance of focusing on the foreground parenchyma region. An ROI attention module can speed up coverage for the background region since the lung volume is always smaller than the rectangular image volume. The proposed deep learning model is able to achieve a 4% higher overall performance and 4.5 folds computational efficiency.

This study is the first attempt to investigate the requirement for a CT to perfusion translation. The key elements for this translation were revealed in this study, which paves the way for more studies on lung function analysis from CT images. In clinical practice, CT based perfusion mapping is advantageous since it has greater availability and does not bring in extra cost. Given the performance of the proposed framework, our method could hold significant values in the diagnosis and therapy of respiratory diseases.

Although the optimized framework was able to estimate the 3D lung perfusion from the CT domain, several factors still need to be considered before clinical implementation. The first consideration is the data heterogeneity. Different medical institutions often have different imaging protocols and equipment, which may affect the robustness of the CT perfusion mapping method. To assess the impact of different protocols, we evaluated the proposed model on nine SPECT/CT scans acquired from a different hospital. The preliminary results showed an average correlation value of 0.6257 ± 0.1566 in the new dataset, decreasing the performance by 4.4%. This indicates re-training on a new dataset may still be necessary at this stage. Besides, different CT scanners are also used in radiation therapy, such as the simulation CT scanner, SPECT/CT scanner, or 4D-CT scanner. Data heterogeneity caused by these scanners may also influence the model performance. Further evaluations on the different imaging protocols and scanners are still warranted in the future.

Another consideration is limited prediction accuracy for some cases. For cases with sharp changing defects, the predicted defect region of CTPM perfusion has higher signals than that in the SPECT perfusion. This phenomenon indicates uncertainty in distinguishing these two kinds of defects. In the following study, we plan to develop a CNN model with more parameters and functional structures to learn features from the sharp changing defect region. Meanwhile, we will collect more SPECT/CT perfusion scans to improve the prediction accuracy and integrate the proposed framework into a computer-aided detection scheme to evaluate disease diagnosis in a clinical setting.

## Conclusions

Our study demonstrates that the deep learning-based CNN model, in conjunction with image processing for feature enhancement, can estimate the perfusion from the CT domain. In the proposed framework, image processing, especially CT contrast enhancement, plays a key role in the perfusion synthesis. The modules and configurations of the CNN model (residual module, ROI attention, skip attention, dropout out, activation function) were also important to improve the prediction performance. This CTPM framework provides insights for relevant research studies in the future. It enables other researchers to leverage the results of this study for the development of optimized CNN models for CTPM.

## Data Availability Statement

The datasets generated for this study are available on request to the corresponding author.

## Ethics Statement

The studies involving human participants were reviewed and approved by the Institutional Review Boards (IRB) of the University of Hong Kong/Hospital Authority Hong Kong West Cluster. The patients/participants provided their written informed consent to participate in this study.

## Author Contributions

GR and JC: carried out primary experiments of project. S-KL, JZ, and HX: provided guidance on overall project and reviewed manuscript. JQ: provided lab and technical support. AC and W-YH: collected data, provided guidance on methodology of the project. All authors contributed to the article and approved the submitted version.

## Funding

This work is supported by the Health and Medical Research Fund (HMRF 07183266); the General Research Fund (GRF 15103520).

## Conflict of Interest

JC, JQ, and W-YH, received funding from Hong Kong Food and Health Bureau (FHB), and Hong Kong University Grants Committee (UGC).

The remaining authors declare that the research was conducted in the absence of any commercial or financial relationships that could be construed as a potential conflict of interest.
