# Corrigendum: Investigation of a novel deep learning-based computed tomography perfusion mapping framework for functional lung avoidance radiotherapy

**DOI:** 10.3389/fonc.2022.1005287

**Published:** 2022-09-05

**Authors:** Ge Ren, Sai-kit Lam, Jiang Zhang, Haonan Xiao, Andy Lai-yin Cheung, Wai-yin Ho, Jing Qin, Jing Cai

**Affiliations:** ^1^ Department of Health Technology and Informatics, The Hong Kong Polytechnic University, Hong Kong, Hong Kong SAR, China; ^2^ Department of Nuclear Medicine, Queen Mary Hospital, Hong Kong, Hong Kong SAR, China; ^3^ School of Nursing, The Hong Kong Polytechnic University, Hong Kong, Hong Kong SAR, China

**Keywords:** perfusion imaging, lung function imaging, deep learning, perfusion synthesis, CT based image analysis, functional lung avoidance radiation therapy

In the published article, there was an error in the Funding statement. The funding statement for the General Research Fund was displayed as “GRF 151022/19M”. The correct Funding statement appears below.

## Funding

This work is supported by the Health and Medical Research Fund (HMRF 07183266); the General Research Fund (GRF 15103520).

The authors apologize for this error and state that this does not change the scientific conclusions of the article in any way. The original article has been updated.

## Publisher’s note

All claims expressed in this article are solely those of the authors and do not necessarily represent those of their affiliated organizations, or those of the publisher, the editors and the reviewers. Any product that may be evaluated in this article, or claim that may be made by its manufacturer, is not guaranteed or endorsed by the publisher.

